# Significance of unphosphorylated and phosphorylated heat shock protein 27 as a prognostic biomarker in pancreatic ductal adenocarcinoma

**DOI:** 10.1007/s00432-020-03175-0

**Published:** 2020-03-21

**Authors:** Richard Drexler, Kim C. Wagner, Mirco Küchler, Bernd Feyerabend, Moritz Kleine, Karl J. Oldhafer

**Affiliations:** 1 Division of Hepatobiliary and Pancreatic Surgery, Department of Surgery, Asklepios Hospital Barmbek, Hamburg, Germany; 2 Asklepios Campus Hamburg, Semmelweis University Budapest, Hamburg, Germany; 3 Department of General, Visceral and Transplant Surgery, Hannover Medical School, Hannover, Germany; 4 MVZ Hanse Histologikum GmbH, Hamburg, Germany

**Keywords:** Heat shock protein, HSP27, Pancreatic cancer, Metastasis, Biomarker, HSP

## Abstract

**Purpose:**

Few studies reported about the potential of unphosphorylated heat shock protein 27 (HSP27) and phosphorylated heat shock protein 27 (pHSP27) as a predictor for survival and gemcitabine resistance in pancreatic ductal adenocarcinoma (PDAC). In this study, we analysed the expression patterns of pHSP27 and HSP27 in a patient population after surgery and correlated the immunohistochemical results with clinicopathological data and long-term outcome of the patients.

**Methods:**

HSP27 and pHSP27 (Ser-15, Ser-78 and Ser-82) protein expression were analysed by immunohistochemistry using the immunoreactive score (IRS) from paraffin-embedded tissue of 106 patients with PDAC who underwent surgery. Immunohistochemical results were correlated with clinicopathological data, disease-free (DFS) and overall survival (OS).

**Results:**

HSP27 expression was significantly lower in patients with a shorter OS (*p* = 0.006) and DFS (*p* < 0.0001). A higher HSP27 expression was associated with a better response to gemcitabine in the resected, non-metastasised patients group (*p* = 0.001). Furthermore, HSP27 was downregulated in patients suffering from metastases at time of surgery (*p* < 0.001) and in undifferentiated tumours (*p* = 0.007). In contrast, pHSP27-Ser15, -Ser78 and -Ser82 were not associated with any survival data of the study population.

**Conclusion:**

HSP27 seems to be a strong indicator for the prediction of OS and DFS. Moreover, HSP27 could play a role in the formation and migration of liver metastases of PDAC.

## Introduction

PDAC remains a challenging disease with a poor overall 5-year survival rate between 4 and 8% (Ilic and Ilic 2016; Ferlay et al. 2013). The best chance for cure with long-term survival is surgical resection. Unfortunately, the majority of the patients display hepatic metastasis at time of diagnosis and only 15–20% are eligible for surgery (Siegel et al. 2018; Vincent et al. 2011).

Heat shock proteins (HSPs) were first discovered by Ritossa et al. as a family of proteins induced by heat shock and other stimuli (Lindquist and Craig 1988; Michel and Starka 1986). These proteins have been characterized as chaperones which are capable of modifying structures of several proteins and can prevent accumulation of misfolded proteins (Ritossa 1996; Gaestel et al. 1989). HSP27 belongs to the small heat shock proteins and can occur in various types depending on post-translational modifications such as phosphorylation at three Serin-sites (Ser-15, Ser-78 and Ser-82) on C-terminal region. The phosphorylation is performed by several kinases including MAPK activated protein kinase 2 (MAPKAPK-2) (Schäfer et al. 1998; Pietersma et al. 1997). Furthermore, several studies report that HSP27 is a major target of protein kinases A, B, C and D (Kostenko and Moens 2009; Gaestel et al. 1991; Butt et al. 2001; Döppler et al. 2005). The effect of phosphorylation is a conformational change of HSP27 which leads to dissociation of large oligomers and the formation of dimers presenting the active leading conformation (Jovcevski et al. 2015).

The phosphorylated and unphosphorylated form of HSP27 has been linked to tumour progression and cell migration (Cayado-Gutiérrez et al. 2013; Zoubeidi and Gleave 2012). Therefore, the expression of HSP27 has been shown to be related to tumour characteristics. Consecutively, the potential of HSP27 in diagnostic, prognostic or treatment implications have been examined in multiple tumour entities. Overexpression of HSP27 correlates with a shorter overall survival (OS) or disease-free survival (DFS) in ovarian, gastric, prostate cancer, and osteosarcoma (Piura et al. 2002; Elpek et al. 2003; Takeno et al. 2001; Bostwick 2000; Uozaki et al. 2000). In contrast, a higher expression is associated with a longer OS in endometrium, oesophageal cancer and malignant fibrous histiocytoma (Geisler et al. 1999; Nakajima et al. 2002; Têtu et al. 1992). These results indicate that HSP27 might serve as a reliable biomarker in human cancer but must be considered separately for every tumour entity (Ciocca and Calderwood 2005).

Only a few clinical studies are available which investigated the potential of HSP27 and pHSP27 to predict survival in PDAC. However, the results are inconsistent or even contradictory (Schäfer et al. 2012; Kawano et al. 2018; Tsiaousidou et al. 2013; Okuno et al. 2016). Therefore, it is still questionable whether HSP27 could be used as a biomarker in PDAC.

Furthermore, weak points of these published studies are reduced numbers of patients and lack of clinical data. Since almost all patients with PDAC receive adjuvant chemotherapy, survival could also be influenced by chemotherapeutic resistance. HSP27 and pHSP27 are shown to be involved in tumour suppression and resistance to chemotherapy with gemcitabine in pancreatic cancer (Guo et al. 2015; Mori-Iwamoto et al. 2007; Kuramitsu et al. 2012; Liu et al. 2012; Taba et al. 2011). pHSP27, which is induced by gemcitabine, is reported to play an important role in the suppression of cancer cell growth in pancreatic cancer cell lines (Taba et al. 2010; Nakashima et al. 2011). However, it is well known that adjuvant therapy with modified FOLFIRINOX results in a longer OS when compared with gemcitabine (Conroy et al. 2018; Yang et al. 2019). Recent evidence indicates that HSP27 could take a significant role in treatment response to mFOLFIRINOX in PDAC. As the mFOLFIRINOX regiment consists of oxaliplatin, irinotecan, 5-fluorouracil and leucovorin, previous studies showed a higher sensitivity for irinotecan and 5-fluorouracil in colon cancer when HSP27 is suppressed (Choi et al. 2007; Hayashi et al. 2012; Shamada et al. 2018). In addition, the inhibition of HSP90 improves the efficacy of oxaliplatin in p53-deficient colon cancer cells (Moser et al. 2007).

In the present study, both HSP27 and pHSP27 were analysed in a patient population diagnosed with PDAC and its expression was correlated with clinicopathological data including OS and DFS.

## Materials and methods

### Ethics approval

All patients’ data were fully anonymised, and the study was performed, according to the standards set in the Declaration of Helsinki 1975. The tumour tissue used was leftover material that had initially been collected for diagnostic purposes. All diagnostic procedures have already been fully completed when samples were retrieved for the study. The study was approved by the Ethics Committee of the Medical Chamber (“Ärztekammer”), Hamburg, Germany (approval number PV5510).

### Patients

A total of 106 patients (female, *n* = 50; male, *n* = 56; median age, 67.2 years [IQR 56.9–76.4 years]) who had been diagnosed with PDAC and went through surgery between 2010 and 2018 were included. Patients’ demographic and clinicopathological characteristics are shown in Table 2. The diagnosis was histologically confirmed and TNM classification was assessed according to AJCC 7th edition. The R-status was obtained pathologically via circumferential resection margin. All patients had a follow-up either until death (*n* = 76) or until their most recent contact (*n* = 28) on August 31, 2019.

### Immunohistochemical analysis

Immunohistochemistry was used to determine the intracellular localization and expression of HSP27 and pHSP27 (Ser-15, Ser-78 and Ser-82). Immunohistochemical staining was performed using paraffin-embedded tissue. Tissue sections (4 µm) were deparaffinized in xylene and rehydrated in a descending alcohol set followed by heated antigen retrieval with 10 mM sodium citrate buffer (pH 6.0) for five minutes. Coverplates™ (ThermoFisher Scientific) were used. Endogenous peroxidase activity was quenched with Peroxide Block (Zytomed Systems). Primary anti-HSP27 monoclonal antibody (working dilution 1:500, Abcam (UK), ab2790) and anti-phosphorylated-HSP27-antibodies (Abcam (UK), Ser15: working dilution 1:350, ab76313; Ser78: working dilution 1:900, ab32501; Ser82: working dilution 1:700, ab90537) were diluted with Antibody Diluent (Zytomed Systems). Sections were covered with antibody and incubated at 4 °C for 24 h. Subsequently, ZytoChem Plus (HRP) Polymer Bulk Kit (Zytomed Systems) were used before staining with DAB (diaminobenzidine) Substrate Kit (Zytomed Systems). Gill’s hematoxylin III (Carl Roth) was used as a counterstaining agent, including a 10 s hydrochloric acid bath (5%) for differentiation. Sections were then dehydrated and mounted with EcoMount (Zytomed Systems).

### Methods of evaluation

An immunoreactive score (IRS) was implemented for the evaluation of the HSP27 expression based on the intensity and quantity of immune staining in pancreatic cancer cells. The IRS score was applied as first described by Kaemmerer et al. (2012) and Remmele and Stegner (1987). The intensity of staining was graded as negative (0), mild (1), moderate (2) and intense (3). The percentage of positive cells was evaluated as 0 (no positive cells), 1 (< 10% positive cells), 2 (10–50% positive cells), 3 (51–80% positive cells) and 4 (> 80% positive cells). The IRS score was obtained by multiplying these two individual scores. As a result, every tissue sample was classified into negative (IRS points 0–1), weak (2–3), mild (4–8) or strong (9–12). Breast carcinoma was used as positive control and brain tissue as negative control. The evaluation of the protein expression was performed by two independent reviewers without knowledge of the patient characteristics.

### Statistical analysis

Differences in continuous variables were analysed with Mann–Whitney *U* test and differences in proportions with chi-square-test or Fisher exact test. DFS and OS was analysed using the Kaplan–Meier method. Univariate and multivariate Cox proportional hazards models were used to assess the effects of variables on OS and DFS. A two-sided p-value less than 0.05 was considered as statistically significant. All analyses were performed using SPSS Inc. (Chicago, IL, USA).

## Results

### Immunohistochemical analysis in PDAC and normal tissue

A positive IRS score of HSP27 with various interindividual intensity was found in the cytoplasm of pancreatic cancer cells in 65 patients (61.3%). Nuclear staining was not detected. Expression of HSP27 was classified into four grades according to the IRS score (negative, mild, moderate and strongly positive; Table 1). 23 patients (21.7%) had a strongly positive staining. We found a significant difference of HSP27 expression between cancer cells and normal pancreatic tissue (*p* < 0.001). In 79 (74.5%) of the corresponding 106 normal pancreatic tissues, HSP27 expression was positive whereof 43 (40.6%) showed a strongly positive staining. In contrast, the majority of patients had a negative IRS score for pHSP27-Ser15 (82 patients, 77.4%) and pHSP27-Ser78 (69 patients, 65.1%). Of the corresponding normal pancreatic tissue, 67 patients (63.2%) were pHSP27-Ser15-negative (*p* = 0.012) and 52 patients (49.1%) pHSP27-Ser78-negative (*p* > 0.001). However, only 48 patients (45.3%) had a negative IRS score for pHSP27-Ser82 in pancreatic cancer cells with 35 patients (33.0%) being negative in corresponding normal pancreatic tissue (*p* > 0.001).

**Table 1:** IRS according to Kaemmerer et al. (2012)

Percentage of positive cells	X intensity of staining	= IRS (0–12)
0 = no positive cells	0 = no colour reaction	0–1 = negative
1 ≤ 10% of positive cells	1 = mild reaction	2–3 = mild
2 = 10–50% positive cells	2 = moderate reaction	4–8 = moderate
3 = 51–80% positive cells	3 = intense reaction	9–12 = strongly positive
4 ≥ 80% positive cells		

IRS-points	IRS-classification
0–1	0 = negative
2–3	1 = positive, weak expression
4–8	2 = positive, mild expression
9–12	3 = positive, strong expression

### Correlation of HSP27 and pHSP27 with clinicopathological features

No significant differences were found between HSP27 expression and gender, age, tumour localization, pathological and lymph node status (Table 2). However, metastasised patients (*n* = 40) showed a significantly lower expression of HSP27 (*p* < 0.001). Of these 40 patients, 32 (80.0%) had a negative IRS score, whereas no patient had a strongly positive score.

**Table 2:** Association between IRS score of unphosphorylated and phosphorylated HSP27 and clinicopathological features of the study population

Characteristic	*n* = 106	HSP27					pHSP27-Ser15					pHSP27-Ser78					pHSP27-Ser82				
		0	1	2	3	*p*	0	1	2	3	*p*	0	1	2	3	*p*	0	1	2	3	*p*
Gender																					
Female	50	14	9	15	12	0.073	36	8	6	0	0.266	30	9	6	5	0.520	18	13	16	3	0.237
Male	56	27	11	7	11		46	7	2	1		39	8	7	2		30	12	10	4	
Tumour localization																					
Head	80	30	13	19	18	0.798	63	10	6	1	0.756	54	9	12	5	**0.016**	36	16	23	5	0.084
Body	7	3	2	0	1		6	0	1	0		2	3	0	2		3	2	1	1	
Tail	11	5	3	1	2		7	4	0	0		9	2	0	0		5	5	0	1	
Body + tail	8	3	2	2	1		6	1	1	0		4	3	1	0		4	2	2	0	
Tumour size^a^																					
< 3.5 cm	45	16	6	10	13	**0.044**	34	6	4	1	0.758	30	4	7	4	0.052	18	11	12	4	0.527
> 3.5 cm	39	17	11	7	4		32	4	3	0		29	8	2	0		18	12	8	1	
Tumour pathological stage																					
T1	7	1	1	2	3	0.491	6	1	0	0	0.675	4	3	0	0	0.271	2	1	2	2	**0.025**
T2	11	6	0	2	3		7	2	2	0		5	3	1	2		5	1	2	3	
T3	76	30	16	14	16		57	12	6	1		50	10	11	5		34	21	20	1	
T4	12	4	3	4	1		12	0	0	0		10	1	1	0		7	2	2	1	
Nodal status																					
N0	24	7	4	5	8	0.738	16	4	4	0	0.421	12	5	4	3	0.468	7	6	7	4	0.091
N1	75	30	15	16	14		59	11	4	1		52	10	9	4		36	19	18	2	
N2	7	4	1	1	1		7	0	0	0		5	2	0	0		5	0	1	1	
Metastasis status																					
M0	66	9	14	20	23	**< 0.001**	48	9	8	1	0.113	41	10	9	6	0.526	26	15	19	6	0.228
M1	40	32	6	2	0		34	6	0	0		28	7	4	1		22	10	7	1	
Tumour differentiation																					
Well-differentiated	7	0	0	2	5	**0.007**	4	3	0	0	0.413	3	2	2	0	0.615	3	1	1	2	0.174
Moderately differentiated	25	6	5	8	6		20	2	3	0		18	4	1	2		8	9	7	1	
Poorly differentiated	67	30	15	10	12		54	8	4	1		42	10	10	5		32	13	18	4	
Anaplastic	7	5	0	2	0		4	2	1	0		6	1	0	0		5	2	0	0	
Resection margin																					
R0	68	20	10	19	19	**0.003**	49	11	7	1	0.296	41	10	10	7	0.126	26	15	20	7	**0.046**
R1	38	21	10	3	4		33	4	1	0		28	7	3	0		22	10	6	0	
Lymphatic invasion																					
L0	40	8	8	11	13	**0.013**	30	6	3	1	0.630	22	6	7	5	0.118	16	9	11	**4**	0.619
L1	66	33	12	11	10		52	9	5	0		47	11	6	2		32	16	15	3	

IRS score is classified as 0 (negative), 1 (positive, weak expression), 2 (positive, mild expression) and 3 (positive, strong expression). All statistically significant variables are highlighted in bold. Statistical analysis: Chi-square test

^a^Data not available for 22 patients (20.7%)

The metastasised subpopulation included 34 patients with liver metastasis (85.0%), 3 patients with peritoneal carcinomatosis (7.5%) and 3 patients with distant lymph node metastasis (7.5%). In contrast, 57 patients (86.4%) within the non-metastasised group expressed HSP27, 23 patients (40.1%) with a strongly positive IRS score.

Furthermore, a lower IRS score for HSP27 was significantly correlated with a bigger tumour size (*p* = 0.044), a less differentiated tumour (*p* = 0.007) and lymphatic invasion (*p* = 0.013). The group of patients with a positive IRS score was more likely to achieve a negative resection margin in surgery (*p* = 0.003).

Immunohistochemical staining of pHSP (Ser-15, Ser-78 and Ser-82) showed a significantly higher IRS score for pHSP27-Ser82 compared to the other sites Ser-78 and Ser-15 in all patients (*p* < 0.001). When correlating the expression of each Serin-site of pHSP27 with clinicopathological characteristics, we observed a significantly lower pHSP-Ser82 expression in patients with a higher pathological T-stage (*p* = 0.025). In addition, the pHSP27-Ser82 expression was significantly associated with a negative resection margin and the pHSP27-Ser78 expression correlated with the tumour localization (*p* = 0.016, Table 2). We could not find a significant correlation between the pHSP27-Ser15 expression and any clinicopathological feature.

### Expression of HSP27 in liver metastasis

26 PDAC patients with synchronous liver metastasis were analysed. Liver metastases as the corresponding primaries showed a negative or weakly positive IRS score for HSP27 expression. Most importantly, we observed similar HSP27 expression in the metastases compared to the primary tumours (*p* = 0.821). None of the examined metastases showed a mildly or strongly positive expression. Similar results were found for all forms of pHSP27.

### HSP27 and long-term outcome

On August 31, 2019, 28 patients were still alive. In 77 patients, data regarding time and type of recurrence were available.

**Fig. 1:**
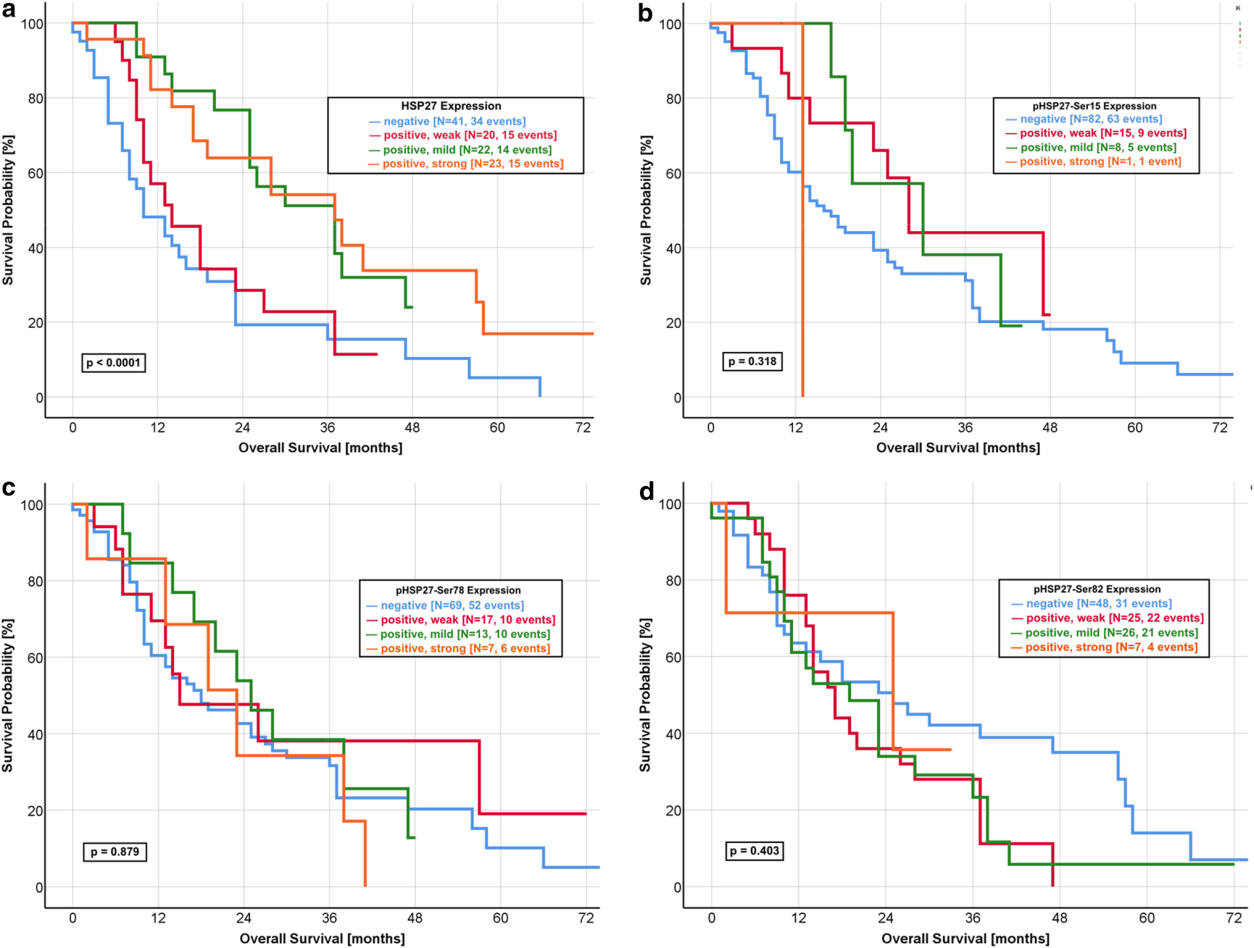
Kaplan–Meier curve of overall survival for HSP27 and pHSP27 expression. **a** HSP27: Strongly and mildly positive expression of HSP27 predicts a favourable OS compared to a negative or weakly positive protein expression (log-rank *p* value < 0.0001). The median OS was 28.2 months in the group with mildly and strongly positive expression. In contrast, the patients group with negative and weakly positive expression had a median OS of 11.0 months. **b** pHSP27-Ser15: The median OS was 16.0 months in patients with a negative expression of pHSP27-Ser15, as compared with 27.4 months for a weakly positive expression and 31.0 months for a mildly positive expression (log-rank *p* value: 0.318). **c** pHSP27-Ser78: No significant difference was found for the OS of pHSP27-Ser78 expression with a comparable median survival (negative: 20.4 months, weakly positive: 25.0 months, mildly positive: 24.6 months, strongly positive: 23.4 months, log-rank *p* value: 0.879). **d** pHSP27-Ser82: The median OS was 24.8 months in patients with a negative expression, as compared with 24.2 months for a strongly positive expression with a non-significant log rank *p* value of 0.403

**Fig. 2:**
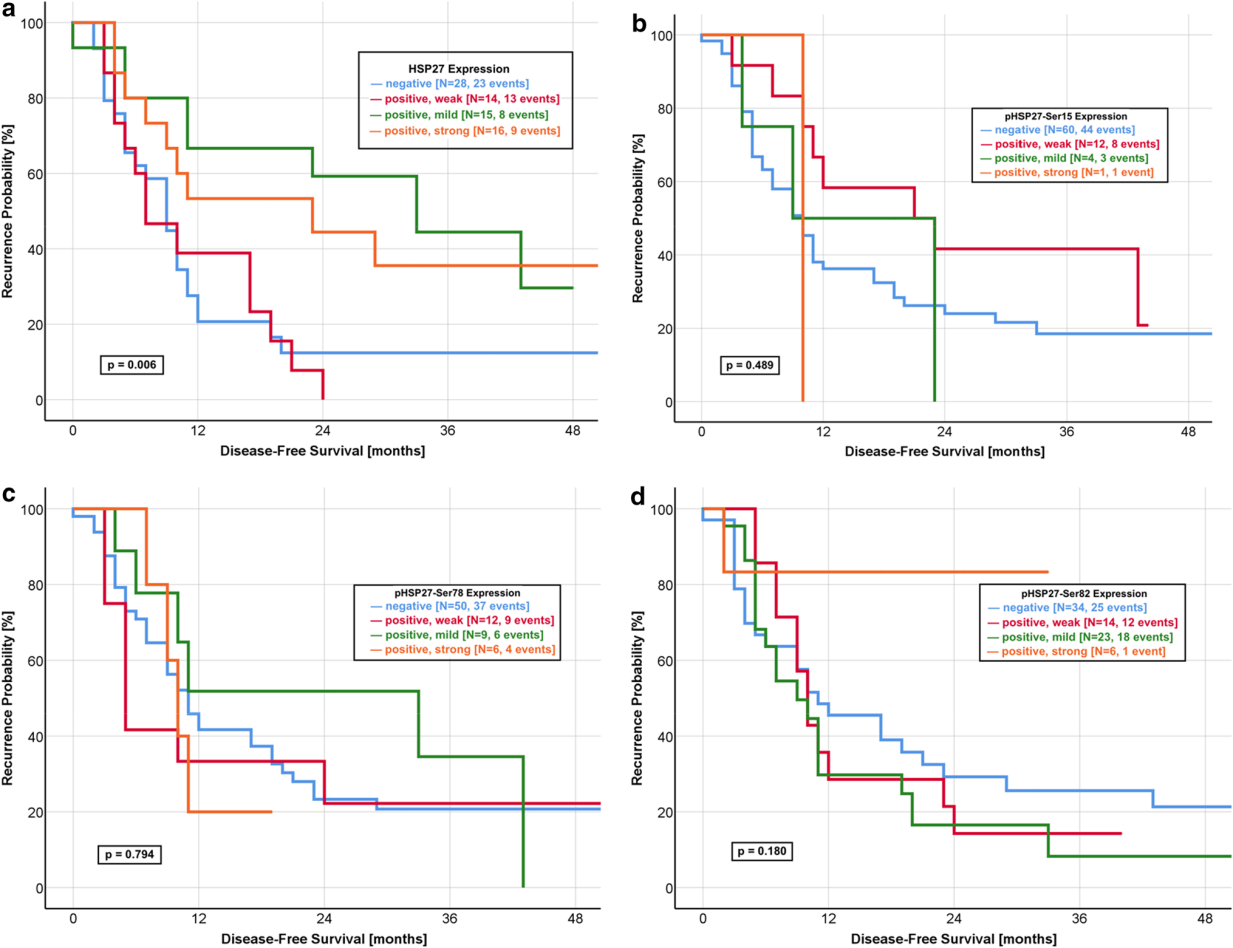
Correlation of HSP27 expression with disease-free survival. **a** HSP27: The median DFS was significantly longer in patients with a strongly (19.0 months) and mildly (25.0 months) positive expression when compared with a negative (9.0 months) and weakly positive (7.0 months) HSP27 expression (log-rank *p* value: 0.006). **b** pHSP27-Ser15: The median DFS was 9.4 months in patients with a negative as well as mildly positive expression of pHSP27-Ser15. Furthermore, the patients group with a weakly positive expression had a median DFS of 21.0 months (log-rank *p* value: 0.489). **c** pHSP27-Ser78: The expression of pHSP27-Ser78 is no predictor for the DFS with a comparable median DFS for all expression groups (negative: 10.0 months, weakly positive: 5.8 months, mildly positive: 11.0 months, strongly positive, 10.0 months, log-rank *p* value: 0.794) **d** pHSP27-Ser82: The median DFS was 11.0 months in patients with a negative expression, as compared with 10.2 months for a strongly positive expression with a non-significant log rank *p* value of 0.180

Using Kaplan–Meier analysis, we found a significant correlation between the HSP27 expression and outcome of the patients. Patients with a mildly or strongly positive HSP27 tumour had a significant better outcome regarding the OS (Fig. 1a, *p* < 0.0001) and DFS (Fig. 2a, *p* = 0.006) than those with a negative or weakly positive expression.

The median OS of patients with a mildly and strongly positive expression was 28.4 months and 28.0 months, respectively. In contrast, patients with HSP27-negative tumours had a median OS of 10.0 months. Nearly similar results were observed for the DFS with a median of 9.0 months in HSP27-negative tumours and 7.0 months in patients with weakly positive HSP27 expression. The median DFS was significantly longer in patients with mildly positive (25.0 months) and strongly positive (19.0 months) HSP27 expression. We could not observe significant differences for any phosphorylated form of HSP27 (Fig. 1b–d, Fig. 2b–d).

We performed multivariate analysis using Cox proportional hazards model to investigate whether the expression of HSP27 or pHSP27 was an independent factor for the outcome after surgery. The results showed that only HSP27 functions as an independent marker for OS (*p* = 0.029, Table 3) and DFS (*p* = 0.015, Table 4). Furthermore, the analysis revealed a significant impact of the tumour pathological stage, resection margin, and vascular invasion for the DFS (Table 4). Interestingly, the patients with HSP27-negative tumours had mainly a different type of recurrence compared to those patients with a positive expression. The group with no expression of HSP27 and a significant shorter DFS formed liver metastasis as type of recurrence in 80.0% of the cases compared to 53.3% in the patients group with a positive HSP27 expression (*p* = 0.029).

**Table 3:** Cox proportional hazard model for overall survival (*n* = 106)

Variable	No	Median OS [months]	Univariate		Multivariate	
			Hazard ratio (95% CI)	*p* value	Hazard ratio (95% CI)	*p* value
HSP27 expression						
Negative	41	10.0	1.00		1.00	
Positive, weak	20	12.1	2.52 (0.87–7.24)	**0.017**	2.99 (1.12–7.99)	**0.029**
Positive, mild	22	28.4	1.57 (0.66–3.75)	0.313	1.98 (0.90–4.37)	**0.048**
Positive, strong	23	28.0	1.01 (0.43–2.45)	0.956	1.01 (0.45–2.28)	0.973
pHSP27-Ser15 expression						
Negative	82	18.0	1.00		1.00	
Positive, weak	15	23.0	1.04 (0.08–13.6)	0.979	1.26 (0.05–31.8)	0.890
Positive, mild	8	22.5	0.54 (0.03–8.6)	0.662	0.69 (0.02–28.5)	0.842
Positive, strong	1	13.0	1.25 (0.07–22.9)	0.879	4.96 (0.12–213.5)	0.404
pHSP27-Ser78 expression						
Negative	69	16.0	1.00		1.00	
Positive, weak	17	14.0	0.71 (0.17–3.1)	0.652	1.94 (0.24–15.6)	0.532
Positive, mild	13	25.0	1.03 (0.14–7.54)	0.975	0.95 (0.08–10.9)	0.966
Positive, strong	7	19.0	0.56 (0.12–2.59)	0.460	0.58 (0.06–5.61)	0.636
pHSP27-Ser82 expression						
Negative	48	21.5	1.00		1.00	
Positive, weak	25	17.0	0.80 (0.14–4.67)	0.807	1.79 (0.16–19.6)	0.635
Positive, mild	26	18.0	1.52 (0.27–8.69)	0.635	7.08 (0.42–118.5)	0.174
Positive, strong	7	19.0	2.29 (0.37–14.4)	0.376	11.7 (0.61–227.1)	0.104
Gender						
Male	56	17.8	1.00		1.00	
Female	50	14.6	1.47 (0.85–2.54)	0.172	1.04 (0.47–2.11)	0.809
Tumour size^a^						
< 3.5 cm	45	19.0	1.00		1.00	
> 3.5 cm	39	11.8	0.83 (0.46–1.50)	0.534	0.84 (0.45–1.59)	0.601
Tumour pathological stage						
T1	7	25.0	1.00		1.00	
T2	11	14.0	0.24 (0.03–1.98)	0.234	0.25 (0.05–1.28)	0.097
T3	76	14.0	0.45 (0.11–1.79)	0.255	0.38 (0.10–1.41)	0.145
T4	12	18.2	0.79 (0.37–1.72)	0.565	0.70 (0.34–1.43)	0.329
Nodal status						
N0	24	23.0	1.00		1.00	
N1	75	16.4	0.44 (0.09–2.0)	0.287	0.453 (0.13–1.26)	0.224
N2	7	9.0	0.91 (0.24–3.39)	0.886	0.747 (0.23–2.44)	0.629
Metastasis status						
M0	66	19.5	1.00		1.00	
M1	40	11.5	0.92 (0.39–2.2)	0.852	1.12 (0.47–2.67)	0.798
Tumour differentiation						
Well-differentiated	7	33.0	1.00		1.00	
Moderately differentiated	25	21.2	1.09 (0.17–6.91)	0.926	1.11 (0.23–5.43)	0.902
Poorly differentiated	67	14.6	1.07 (0.29–3.88)	0.923	0.98 (0.45–3.39)	0.983
Anaplastic	7	9.2	1.36 (0.41–4.55)	0.613	1.24 (0.45–3.39)	0.681
Resection margin						
R0	68	18.0	1.00		1.00	
R1	38	14.0	0.66 (0.34–1.29)	0.230	1.19 (0.67–2.14)	0.551
Lymphatic invasion						
L0	40	24.2	1.00		1.00	
L1	66	14.0	1.15 (0.62–2.13)	0.721	1.21 (0.61–2.39)	0.584
Perineural invasion						
Pn0	9	25.0	1.00		1.00	
Pn1	97	16.0	0.81 (0.28–2.34)	0.693	1.40 (0.55–3.60)	0.480
Vascular invasion						
V0	50	20.5	1.00		1.00	
V1	56	14.0	1.15 (0.62–2.13)	0.658	0.68 (0.39–1.19)	0.184

*CI* confidence interval. All statistically significant variables are highlighted in bold

^a^Data not available for 22 patients (20.7%)

**Table 4:** Cox proportional hazard model for disease-free survival (*n* = 77)

Variable	No	Median DFS [months]	Univariate		Multivariate	
			Hazard ratio (95% CI)	*p* value	Hazard ratio (95% CI)	*p* value
HSP27 expression						
Negative	31	9.0	1.00		1.00	
Positive, weak expression	15	7.0	2.84 (0.79–10.1)	**0.017**	5.55 (1.39–22.0)	**0.015**
Positive, mild expression	15	25.0	2.89 (0.88–9.59)	**0.042**	3.67 (1.04–12.9)	**0.044**
Positive, strong expression	16	19.0	1.46 (0.45–4.79)	0.533	2.88 (0.78–10.7)	0.113
pHSP27-Ser15 expression						
Negative	60	9.0	1.00		1.00	
Positive, weak expression	12	22.0	0.96 (0.07–12.8)	0.974	0.70 (0.02–27.4)	0.850
Positive, mild expression	4	14.0	0.27 (0.02–4.98)	0.379	0.27 (0.01–21.4)	0.560
Positive, strong expression	1	10.0	0.47 (0.02–9.85)	0.627	0.36 (0.01–15.3)	0.592
pHSP27-Ser78 expression						
Negative	50	10.5	1.00		1.00	
Positive, weak expression	12	8.0	0.86 (0.11–6.35)	0.875	1.35 (0.05–33.2)	0.856
Positive, mild expression	9	11.0	0.85 (0.09–8.4)	0.888	0.99 (0.03–30.9)	0.995
Positive, strong expression	6	9.5	1.12 (0.14–10.4)	0.872	6.23 (0.29–132.7)	0.241
pHSP27-Ser82 expression						
Negative	34	10.5	1.00		1.00	
Positive, weak expression	14	10.0	1.28 (0.07–25.2)	0.873	2.88 (0.12–67.7)	0.521
Positive, mild expression	23	8.0	1.75 (0.09–35.3)	0.715	1.95 (0.55–69.1)	0.713
Positive, strong expression	6	11.0	1.09 (0.68–1.77)	0.689	1.85 (0.06–53.1)	0.721
Gender						
Male	44	9.5	1.00		1.00	
Female	33	11.0	1.12 (0.49–2.55)	0.789	0.91 (0.36–2.31)	0.844
Tumour size						
< 3.5 cm	29	11.0	1.00		1.00	
> 3.5 cm	30	7.5	2.39 (0.98–5.86)	0.055	0.66 (0.26–1.71)	0.392
Tumour pathological stage						
T1	6	21.5	1.00		1.00	
T2	10	10.2	0.19 (0.03–1.48)	0.113	0.06 (0.07–0.63)	**0.019**
T3	55	9.0	0.49 (0.09–2.52)	0.394	0.15 (0.2–1.26)	**0.08**
T4	6	11.5	0.58 (0.17–2.03)	0.394	0.42 (0.11–1.65)	0.215
Nodal status						
N0	16	19.0	1.00		1.00	
N1	54	9.5	0.14 (0.02–0.94)	**0.043**	0.18 (0.03–1.16)	0.071
N2	7	3.0	0.12 (0.04–0.37)	**0.001**	0.08 (0.02–0.35)	**0.001**
Metastasis status						
M0	49	11.0	1.00		1.00	
M1	28	9.0	0.48 (0.17–1-37)	0.167	2.31 (0.75–7.07)	0.143
Tumour differentiation						
Well differentiated	4	32.5	1.00		1.00	
Moderately differentiated	19	12.0	1.47 (0.06–35.7)	0.814	0.70 (0.02–21.4)	0.840
Poorly differentiated	51	9.0	6.79 (0.59–77.3)	0.123	7.49 (0.59–95.1)	0.120
Anaplastic	3	1.0	7.16 (0.71–72.1)	0.095	7.70 (0.73–81.8)	0.090
Resection margin						
R0	50	19.0	1.00		1.00	
R1	27	10.5	0.31 (0.14–0.68)	**0.004**	4.64 (1.62–13.3)	**0.004**
Lymphatic invasion						
L0	28	13.5	1.00		1.00	
L1	49	9.0	0.41 (0.13–1.29)	0.126	3.28 (0.92–11.7)	0.067
Perineural invasion						
Pn0	6	22.0	1.00		1.00	
Pn1	71	14.5	0.56 (0.82–3.90)	0.562	0.13 (0.01–1.89)	0.136
Vascular invasion						
V0	33	17.0	1.00		1.00	
V1	44	8.0	2.63 (0.89–7.77)	0.080	0.27 (0.09–0.84)	**0.023**

*CI* confidence interval. All statistically significant variables are highlighted in bold

### HSP27 as a predictor for gemcitabine sensitivity

**Fig. 3:**
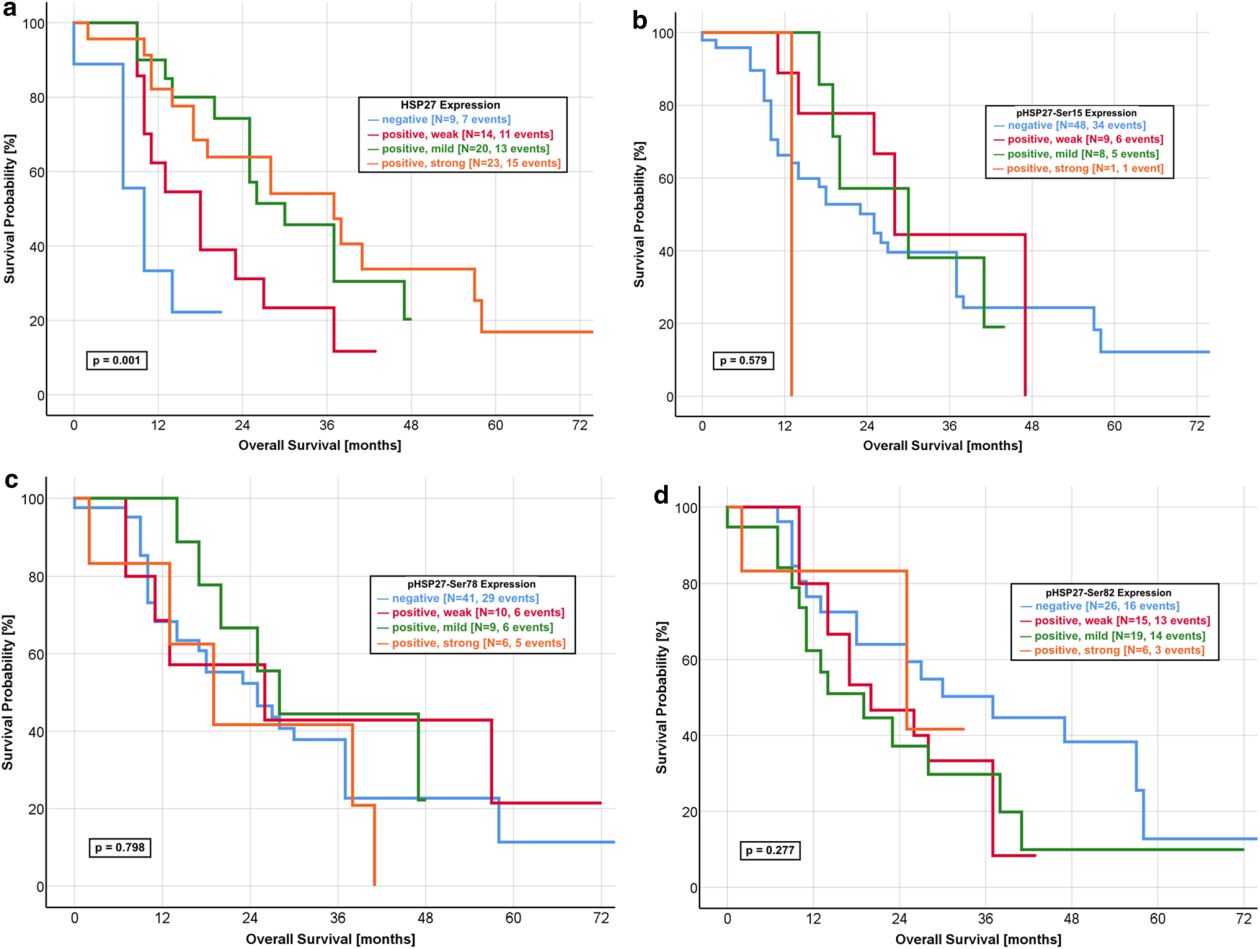
Kaplan–Meier curves for overall survival in the gemcitabine-treated subpopulation. **a** HSP27: Strongly and mildly positive expression of HSP27 predicts a favourable OS in the non-metastasised and gemcitabine-treated patients, as compared to a negative or weakly positive protein expression (log-rank *p* value: 0.001). The median OS was 37.0 months in the group with a strongly positive expression and 30.0 months for a mildly positive expression. In contrast, the patients group with a negative expression had a median OS of 10.0 months. **b** pHSP27-Ser15: The patients group with a negative expression of pHSP27-Ser15 had a median OS of 25.0 months. A comparable median OS was found in patients with a weakly positive (27.2 months) and mildly positive (29.0 months) expression (log-rank *p* value: 0.579). **c** pHSP27-Ser78: No significant difference was found for the OS of pHSP27-Ser78 expression with a comparable median survival (negative: 25.0 months, weakly positive: 25.4 months, mildly positive: 27.4 months, strongly positive: 19.0 months, log-rank *p* value: 0.798). **d** pHSP27-Ser82: The median OS was 30.2 months in patients with a negative expression, as compared with 25.0 months for a strongly positive expression with a non-significant log rank *p* value of 0.277

We created a subpopulation of 66 resected, non-metastasised and gemcitabine-treated patients. In this patients group, we investigated the influence of the HSP27 expression on the OS (Fig. 3a–d). Kaplan–Meier analysis revealed a significant better outcome of patients with a mildly or strongly positive HSP27 expression compared to those with a negative or weakly positive IRS score (*p* = 0.001). In addition, we applied Kaplan–Meier analysis for all forms of pHSP27 (Ser-15, Ser-78 and Ser-82) but could not find any significant differences (Fig. 3b, c).

## Discussion

Previous studies investigated the potential of HSP27 and pHSP27 as a prognostic biomarker in several tumour entities and found different results (Love and King 1994; Têtu et al. 1995; Eto et al. 2016; Elpek et al. 2003; Takeno et al. 2001; Uozaki et al. 2000). Therefore, HSP27 and pHSP27 expression and their role as a prognostic biomarker should be investigated separately for each cancer type.

Particularly for PDAC, only three clinical studies are available in the literature. Schäfer et al. (2012) and Okuno et al. (2016) used both the unphosphorylated and phosphorylated (-Ser82) form of HSP27. However, Schäfer et al. investigated the protein expression in tissue specimens from patients who underwent surgery, while Okuno et al. used biopsied pancreatic cancer tissue. Another study by Tsiaousidou et al. (2013) analysed only the unphosphorylated form of HSP27. To our knowledge, our study is the largest with 106 patients and the only one considering the unphosphorylated as well as all phosphorylated (Ser-15, Ser-78, Ser-82) forms of HSP27.

The aforementioned study by Schäfer et al. (2012) found a significant longer OS in 86 patients with a higher expression of unphosphorylated form of HSP27. In addition, Okuno et al. (2016) correlated a higher pHSP27-Ser82 expression with a longer OS in 49 patients. Our study, does confirm the findings of the study by Schäfer et al. as we found a significant longer OS in patients with mildly or strongly positive HSP27 expression. However, we could not find any significant correlation between the expression of pHSP27 and outcome of the patients.

Furthermore, our study revealed a significant shorter DFS of patients with negative or weakly positive expression of the unphosphorylated HSP27 and multivariate analyses determined the HSP27 expression as an independent factor for DFS. Taking a closer look at the patients group suffering from a recurrence shows that patients with HSP27-negative tumours had a significantly higher incidence of liver metastases as a type of recurrence. To our knowledge, no study ever before had investigated the link between HSP27 and time as well as type of recurrence.

Therefore, the HSP27 expression seems to have the potential to predict not only the long-term outcome regarding OS and DFS but also the type of recurrence. Combining these results with those of Schäfer et al. concerning the findings for the OS it is obvious that tumours with a lower HSP27 expression are accompanied with a poor outcome of patients with PDAC.

One reason for a longer survival of patients with HSP27-positive tumours could be a higher sensitivity against adjuvant chemotherapy with gemcitabine.

Mori-Iwamato et al. (2007) investigated HSP27 as one of the target proteins of gemcitabine using proteomic analysis. Furthermore, there is evidence for pancreatic cancer cell lines that overexpression of HSP27 enhanced the sensitivity towards gemcitabine (Guo et al. 2015). While Guo et al. found the unphosphorylated form of HSP27 as an essential component for gemcitabine sensitivity, another study by Kang et al. (2015) described the ratio of phosphorylated to non-phosphorylated HSP27 as more important than the cellular level of HSP27 itself. In addition, Nakashima et al. (2011) suggest the phosphorylation status of HSP27 as an essential determinant for gemcitabine-induced suppression of pancreatic cancer cells. In our study, we investigated a subpopulation of 66 non-metastasised and resected patients which were treated with gemcitabine as adjuvant chemotherapy. The Kaplan–Meier analysis clearly shows a worse survival in patients with negative or weakly positive HSP27 expression compared to those with a mildly or strongly positive protein expression. Similar results could already be shown by Schäfer et al. (2012) where a negative HSP27 expression was associated with a shorter OS in gemcitabine-treated patients. Therefore, the influence of HSP27 towards the sensitivity of gemcitabine has a huge impact on the survival of resected patients. Even though Nakashima et al. and Kang et al. reported about the significance of phosphorylated forms of HSP27 regarding the effect of gemcitabine, our study does not show a significant correlation between pHSP27 and survival in the gemcitabine-treated subpopulation.

Another finding of our study was the downregulation of HSP27 in patients suffering from simultaneous metastases. The downregulation was also found in their relating liver metastases. These results indicate a possible role of HSP27 in the formation of liver metastases. Taken together with the correlation of a low HSP27 expression and liver metastases as a type of an early disease recurrence, we hypothesize that the downregulation of HSP27 could be one of the underlying mechanisms for the migration of pancreatic cancer cells in the liver. Previous studies already tried to investigate the specific impact of HSP27 in formation of metastasis of different cancer types. It is reported that HSP27 can increase transforming growth factor b (TGF-b)-stimulated MMP2 activity and therefore promotes cell invasion in prostate cancer (Xu et al. 2006). In addition, HSP27 modulates the expression of phosphatase and tensin homolog (PTEN) by which the PI3-kinase pathway is influenced in breast cancer (Cayado-Gutiérrez et al. 2013). Furthermore, Vahid et al. (2016) revealed an interaction between HSP27 and the pro-metastatic Hippo pathway in prostate, breast and lung cancer but the impact of this regulation on migration of metastasis needs to be elucidated. At this time, we do not understand the specific mechanisms how the activity and expression of HSP27 is regulated during the formation of metastasis and, consequently which targets are influenced (Wu et al. 2017). Nevertheless, our results show that HSP27 could act as an important factor in metastatic invasion which comes along with a poor outcome of patients with PDAC.

## Conclusion

To conclude, our study demonstrates that a downregulation of HSP27 is associated with a poor outcome for patients with PDAC and therefore HSP27 could serve as a predictor for the OS and DFS. Furthermore, patients with stronger HSP27 expression seem to have a higher sensitivity for gemcitabine resulting in a longer OS. Beside this, patients with a negative HSP27 expression were either presenting simultaneous liver metastasis at time of surgery or developed mainly liver metastasis after surgical resection of the primary tumour within a short time. Therefore, the impact of HSP27 in formation of liver metastasis is obvious but the underlying mechanisms and involvement of specific pathways need to be elucidated in further studies.

## References

[CR1] Bostwick DG (2000) Immunohistochemical changes in prostate cancer after androgen deprivation therapy. Mol Urol 4(3):101–106 **(discussion 107)** 11062363

[CR2] Butt E, Immler D, Meyer HE, Kotlyarov A, Laass K, Gaestel M (2001) Heat shock protein 27 Is a substrate of CGMP-dependent protein kinase in intact human platelets: phosphorylation-induced actin polymerization caused by HSP27 mutants. J Biol Chem 276(10):7108–7113 11383510 10.1074/jbc.m009234200

[CR3] Cayado-Gutiérrez N, Moncalero VL, Rosales EM, Berón W, Salvatierra EE, Alvarez-Olmedo D, Radrizzani M, Ciocca DR (2013) Downregulation of Hsp27 (HSPB1) in MCF-7 human breast cancer cells induces upregulation of PTEN. Cell Stress Chaperones 18(2):243–249. 10.1007/s12192-012-0367-x 22907762 10.1007/s12192-012-0367-x PMC3581620

[CR4] Choi DH, Ha JS, Lee WH, Song JK, Kim GY, Park JH, Cha HJ, Lee BJ, Park JW (2007) Heat shock protein 27 is associated with irinotecan resistance in human colorectal cancer cells. FEBS Lett 581(2007):1649–1656. 10.1016/j.febslet.2007.02.075 17395183 10.1016/j.febslet.2007.02.075

[CR5] Ciocca DR, Calderwood SK (2005) Heat shock proteins in cancer: diagnostic, prognostic, predictive, and treatment implications. Cell Stress Chaperones 10(2):86–103 16038406 10.1379/CSC-99r.1 PMC1176476

[CR6] Conroy T, Hammel P, Hebbar M, Ben Abdelghani M, Wei AC, Raoul J-L et al (2018) FOLFIRINOX or gemcitabine as adjuvant therapy for pancreatic cancer. N Engl J Med 379(25):2395–2406. 10.1056/NEJMoa1809775 30575490 10.1056/NEJMoa1809775

[CR7] Döppler H, Storz P, Li J, Comb MJ, Toker A (2005) A phosphorylation state-specific antibody recognizes Hsp27, a novel substrate of protein kinase D. J Biol Chem 280(15):15013–15019. 10.1074/jbc.C400575200 15728188 10.1074/jbc.C400575200

[CR8] Elpek GO, Karaveli S, Simşek T, Keles N, Aksoy NH (2003) Expression of heat-shock proteins Hsp27, Hsp70 and Hsp90 in malignant epithelial tumour of the ovaries. APMIS Acta Pathol Microbiol Immunol Scand 111(4):523–530 10.1034/j.1600-0463.2003.1110411.x 12780528

[CR9] Eto D, Hisaka T, Horiuchi H, Uchida S, Ishikawa H, Kawashima Y, Kinugasa T et al (2016) Expression of HSP27 in hepatocellular carcinoma. Anticancer Res 36(7):3775–3779 27354654

[CR10] Ferlay J, Steliarova-Foucher E, Lortet-Tieulent J, Rosso S, Coebergh JWW, Comber H, Forman D, Bray F (2013) Cancer incidence and mortality patterns in Europe: estimates for 40 countries in 2012. Eur J Cancer 49(6):1374–1403. 10.1016/j.ejca.2012.12.027 23485231 10.1016/j.ejca.2012.12.027

[CR11] Gaestel M, Gross B, Benndorf R, Strauss M, Schunk WH, Kraft R, Otto A, Böhm H, Stahl J, Drabsch H (1989) Molecular cloning, sequencing and expression in *Escherichia Coli* of the 25-KDa growth-related protein of ehrlich ascites tumor and its homology to mammalian stress proteins. Eur J Biochem 179(1):209–213 2645135 10.1111/j.1432-1033.1989.tb14542.x

[CR12] Gaestel M, Schröder W, Benndorf R, Lippmann C, Buchner K, Hucho F, Erdmann VA, Bielka H (1991) Identification of the phosphorylation sites of the murine small heat shock protein Hsp25. J Biol Chem 266(22):14721–14724 1860870

[CR13] Geisler JP, Geisler HE, Tammela J, Miller GA, Wiemann MC, Zhou Z (1999) A study of heat shock protein 27 in endometrial carcinoma. Gynecol Oncol 72(3):347–350. 10.1006/gyno.1998.5283 10053106 10.1006/gyno.1998.5283

[CR14] Guo Y, Ziesch A, Hocke S, Kampmann E, Ochs S, De Toni EN, Göke B, Gallmeier E (2015) Overexpression of heat shock protein 27 (HSP27) increases gemcitabine sensitivity in pancreatic cancer cells through S-phase arrest and apoptosis. J Cell Mol Med 19(2):340–350. 10.1111/jcmm.12444 25331547 10.1111/jcmm.12444 PMC4407596

[CR15] Hayashi R, Ishii Y, Ochiai H, Matsunaga A, Endo T, Kitagawa Y (2012) Suppression of heat shock protein 27 expression promotes 5-fluorouracil sensitivity in colon cancer cells in a xenograft model. Oncol Rep 28:1269–1274. 10.3892/or.2012.1935 22842517 10.3892/or.2012.1935

[CR16] Ilic M, Ilic I (2016) Epidemiology of pancreatic cancer. World J Gastroenterol 22(44):9694–9705. 10.3748/wjg.v22.i44.9694 27956793 10.3748/wjg.v22.i44.9694 PMC5124974

[CR17] Jovcevski B, Kelly MA, Rote AP, Berg T, Gastall HY, Justin LP, Benesch J, Aquilina A, Ecroyd H (2015) Phosphomimics destabilize Hsp27 oligomeric assemblies and enhance chaperone activity. Chem Biol 22(2):186–195. 10.1016/j.chembiol.2015.01.001 25699602 10.1016/j.chembiol.2015.01.001

[CR18] Kaemmerer D, Peter L, Lupp A, Schulz S, Sänger J, Baum RP, Prasad V, Hommann M (2012) Comparing of IRS and Her2 as immunohistochemical scoring schemes in gastroenteropancreatic neuroendocrine tumors. Int J Clin Exp Pathol 5(3):187–194 22558472 PMC3341681

[CR19] Kang D, Choi HJ, Kang S, Kim SY, Hwang Y-S, Je S, Han Z, Kim J-H, Song JJ (2015) Ratio of phosphorylated HSP27 to nonphosphorylated HSP27 biphasically acts as a determinant of cellular fate in gemcitabine-resistant pancreatic cancer cells. Cell Signal 27(4):807–817. 10.1016/j.cellsig.2015.01.007 25615626 10.1016/j.cellsig.2015.01.007

[CR20] Kawano M, Kaino S, Amano S, Shinoda S, Suenaga S, Sen-Yo M, Sakaida I (2018) Heat shock protein 27 expression in EUS-FNA samples can predict gemcitabine sensitivity in pancreatic cancer. In Vivo 32(3):637–642. 10.21873/invivo.11286 29695571 10.21873/invivo.112286 PMC6000801

[CR21] Kostenko S, Moens U (2009) Heat shock protein 27 phosphorylation: kinases, phosphatases, functions and pathology. Cell Mol Life Sci CMLS 66(20):3289–3307. 10.1007/s00018-009-0086-3 19593530 10.1007/s00018-009-0086-3 PMC11115724

[CR22] Kuramitsu Y, Wang Y, Taba K, Suenaga S, Ryozawa S, Kaino S, Sakaida I, Nakamura K (2012) Heat-shock protein 27 plays the key role in gemcitabine-resistance of pancreatic cancer cells. Anticancer Res 32(6):2295–2299 22641665

[CR23] Lindquist S, Craig EA (1988) The heat-shock proteins. Annu Rev Genet 22:631–677. 10.1146/annurev.ge.22.120188.003215 2853609 10.1146/annurev.ge.22.120188.003215

[CR24] Liu Q-H, Zhao C-Y, Zhang J, Chen Y, Gao Li, Ni C-Y, Zhu M-H (2012) Role of heat shock protein 27 in gemcitabine-resistant human pancreatic cancer: comparative proteomic analyses. Mol Med Rep 6(4):767–773. 10.3892/mmr.2012.1013 22858734 10.3892/mmr.2012.1013

[CR25] Love S, King RJ (1994) A 27 KDa heat shock protein that has anomalous prognostic powers in early and advanced breast cancer. Br J Cancer 69(4):743–748 8142264 10.1038/bjc.1994.140 PMC1968803

[CR26] Michel GP, Starka J (1986) Effect of ethanol and heat stresses on the protein pattern of *Zymomonas Mobilis*. J Bacteriol 165(3):1040–1042 3949711 10.1128/jb.165.3.1040-1042.1986 PMC214537

[CR27] Mori-Iwamoto S, Kuramitsu Y, Ryozawa S, Mikuria K, Fujimoto M, Maehara S-I, Maehara Y, Okita K, Nakamura K, Sakaida I (2007) Proteomics finding heat shock protein 27 as a biomarker for resistance of pancreatic cancer cells to gemcitabine. Int J Oncol 31(6):1345–1350 17982661

[CR28] Moser C, Lang SA, Kainz S, Gaumann A, Fichtner-Feigl S, Koehl GE, Schlitt HJ, Geissler EK, Stoeltzing O (2007) Blocking heat shock protein-90 inhibits the invasive properties and hepatic growth of human colon cancer cells and improves the efficacy of oxaliplatin in p53-deficient colon cancer tumors in vivo. Mol Cancer Ther 6(11):2868–2878. 10.1158/1535-7163.MCT-07-0410 18025273 10.1158/1535-7163.MCT-07-0410

[CR29] Nakajima M, Kuwano H, Miyazaki T, Masuda N, Kato H (2002) Significant correlation between expression of heat shock proteins 27, 70 and lymphocyte infiltration in esophageal squamous cell carcinoma. Cancer Lett 178(1):99–106 11849747 10.1016/s0304-3835(01)00825-4

[CR30] Nakashima M, Adachi S, Yasuda I, Yamauchi T, Kawaguchi J, Itani M, Yoshioka T et al (2011) Phosphorylation status of heat shock protein 27 plays a key role in gemcitabine-induced apoptosis of pancreatic cancer cells. Cancer Lett 313(2):218–225. 10.1016/j.canlet.2011.09.008 21999932 10.1016/j.canlet.2011.09.008

[CR31] Okuno M, Yasuda I, Adachi S, Nakashima M, Kawaguchi J, Doi S, Iwashita T et al (2016) The significance of phosphorylated heat shock protein 27 on the prognosis of pancreatic cancer. Oncotarget 7(12):14291–14299. 10.18632/oncotarget.7424 26895107 10.18632/oncotarget.7424 PMC4924715

[CR32] Pietersma A, Tilly BC, Gaestel M, de Jong N, Lee JC, Koster JF, Sluiter W (1997) P38 mitogen activated protein kinase regulates endothelial VCAM-1 expression at the post-transcriptional level. Biochem Biophys Res Commun 230(1):44–48. 10.1006/bbrc.1996.5886 9020057 10.1006/bbrc.1996.5886

[CR33] Piura B, Rabinovich A, Yavelsky V, Wolfson M (2002) Heat shock proteins and malignancies of the female genital tract. Harefuah 141(11):969–972 12476632

[CR34] Remmele W, Stegner HE (1987) Recommendation for uniform definition of an immunoreactive score (IRS) for immunohistochemical estrogen receptor detection (ER-ICA) in breast cancer tissue. Der Pathol 3(8):138–140 3303008

[CR35] Ritossa F (1996) Discovery of the heat shock response. Cell Stress Chaperones 1(2):97–98 9222594 10.1379/1466-1268(1996)001<0097:dothsr>2.3.co;2 PMC248460

[CR36] Schäfer C, Ross SE, Bragado MJ, Groblewski GE, Ernst SA, Williams JA (1998) A role for the P38 mitogen-activated protein kinase/Hsp 27 pathway in cholecystokinin-induced changes in the actin cytoskeleton in rat pancreatic acini. J Biol Chem 273(37):24173–24180 9727040 10.1074/jbc.273.37.24173

[CR37] Schäfer C, Seeliger H, Bader DC, Assmann G, Buchner D, Guo Y, Ziesch A et al (2012) Heat shock protein 27 as a prognostic and predictive biomarker in pancreatic ductal adenocarcinoma. J Cell Mol Med 16(8):1776–1791. 10.1111/j.1582-4934.2011.01473.x 22004109 10.1111/j.1582-4934.2011.01473.x PMC3822691

[CR38] Shamada T, Tsuruta M, Hasegawa H, Okabayashi K, Shigeta K et al (2018) Heat shock protein 27 knockdown using nucleotide-based therapies enhances sensitivity to 5-FU chemotherapy in SW480 human colon cancer cells. Oncol Rep 39:1119–1124. 10.3892/or.2018.6180 29328475 10.3892/or.2018.6180

[CR39] Siegel RL, Miller KD, Jemal A (2018) Cancer statistics, 2018. CA Cancer J Clin 68(1):7–30. 10.3322/caac.21442 29313949 10.3322/caac.21442

[CR40] Taba K, Kuramitsu Y, Ryozawa S, Yoshida K, Tanaka T, Maehara S-I, Maehara Y, Sakaida I, Nakamura K (2010) Heat-shock protein 27 is phosphorylated in gemcitabine-resistant pancreatic cancer cells. Anticancer Res 30(7):2539–2543 20682980

[CR41] Taba K, Kuramitsu Y, Ryozawa S, Yoshida K, Tanaka T, Mori-Iwamoto S, Maehara S-I, Maehara Y, Sakaida I, Nakamura K (2011) KNK437 downregulates heat shock protein 27 of pancreatic cancer cells and enhances the cytotoxic effect of gemcitabine. Chemotherapy 57(1):12–16. 10.1159/000321019 21124027 10.1159/000321019

[CR42] Takeno S, Noguchi T, Kikuchi R, Sato T, Uchida Y, Yokoyama S (2001) Analysis of the survival period in resectable stage IV gastric cancer. Ann Surg Oncol 8(3):215–221 11314937 10.1007/s10434-001-0215-1

[CR43] Têtu B, Brisson J, Landry J, Huot J (1995) Prognostic significance of heat-shock protein-27 in node-positive breast carcinoma: an immunohistochemical study. Breast Cancer Res Treat 36(1):93–97 7579511 10.1007/BF00690189

[CR44] Têtu B, Lacasse B, Bouchard HL, Lagacé R, Huot J, Landry J (1992) Prognostic influence of HSP-27 expression in malignant fibrous histiocytoma: a clinicopathological and immunohistochemical study. Can Res 52(8):2325–2328 1313743

[CR45] Tsiaousidou A, Lambropoulou M, Chatzitheoklitos E, Tripsianis G, Tsompanidou C, Simopoulos C, Tsaroucha AK (2013) B7H4, HSP27 and DJ-1 molecular markers as prognostic factors in pancreatic cancer. Pancreatology 13(6):564–569. 10.1016/j.pan.2013.10.005 24280570 10.1016/j.pan.2013.10.005

[CR46] Uozaki H, Ishida T, Kakiuchi C, Horiuchi H, Gotoh T, Iijima T, Imamura T, Machinami R (2000) Expression of heat shock proteins in osteosarcoma and its relationship to prognosis. Pathol Res Pract 196(10):665–673. 10.1016/S0344-0338(00)80118-1 11087053 10.1016/S0344-0338(00)80118-1

[CR47] Vahid S, Thaper D, Gibson KF, Bishop JL, Zoubeidi A (2016) Molecular chaperone Hsp27 regulates the Hippo tumour suppressor pathway in cancer. Nat Sci Rep. 10.1038/srep31842 10.1038/srep31842 PMC4995483 27555231

[CR48] Vincent A, Herman J, Schulick R, Hruban RH, Goggins M (2011) Pancreatic cancer. Lancet 378(9791):607–620. 10.1016/S0140-6736(10)62307-0 21620466 10.1016/S0140-6736(10)62307-0 PMC3062508

[CR49] Wu J, Liu T, Rios Z, Mei Q, Lin X, Cao S (2017) Heat shock proteins and cancer. Trends Pharmacol Sci 38(3):226–256. 10.1016/j.tips.2016.11.009 28012700 10.1016/j.tips.2016.11.009

[CR50] Xu L, Chen S, Bergan RC (2006) MAPKAPK2 and HSP27 are downstream effectors of P38 MAP kinase-mediated matrix metalloproteinase type 2 activation and cell invasion in human prostate cancer. Oncogene 25(21):2987–2998. 10.1038/sj.onc.1209337 16407830 10.1038/sj.onc.1209337

[CR51] Yang F, Jin C, Fu DL, Warshaw AL (2019) Modified FOLFIRINOX for resected pancreatic cancer: opportunities and challenges. World J Gastroenterol 25(23):2839–2845. 10.3748/wjg.v25.i23.2839 31249443 10.3748/wjg.v25.i23.2839 PMC6589737

[CR52] Zoubeidi A, Gleave M (2012) Small heat shock proteins in cancer therapy and prognosis. Int J Biochem Cell Biol 44(10):1646–1656. 10.1016/j.biocel.2012.04.010 22571949 10.1016/j.biocel.2012.04.010

